# Fulfilling the Promise of RNA Therapies for Cardiac Repair and Regeneration

**DOI:** 10.1093/stcltm/szad038

**Published:** 2023-07-13

**Authors:** Mauro Giacca

**Affiliations:** School of Cardiovascular and Metabolic Medicine & Sciences and British Heart Foundation Centre of Research Excellence, King’s College London, London, UK; Department of Medical Sciences, University of Trieste, Italy

**Keywords:** MicroRNA, siRNA, mRNA, cardiac regeneration, heart, angiogenesis, gene editing, lipid nanoparticles, cardioprotection

## Abstract

The progressive appreciation that multiple types of RNAs regulate virtually all aspects of tissue function and the availability of effective tools to deliver RNAs in vivo now offers unprecedented possibilities for obtaining RNA-based therapeutics. For the heart, RNA therapies can be developed that stimulate endogenous repair after cardiac damage. Applications in this area include acute cardioprotection after ischemia or cancer chemotherapy, therapeutic angiogenesis to promote new blood vessel formation, regeneration to form new cardiac mass, and editing of mutations to cure inherited cardiac disease. While the potential of RNA therapeutics for all these conditions is exciting, the field is still in its infancy. A number of roadblocks need to be overcome for RNA therapies to become effective, in particular, related to the problem of delivering RNA medicines into the cells and targeting them specifically to the heart.

## Graphical abstract

**Graphical Abstract:**
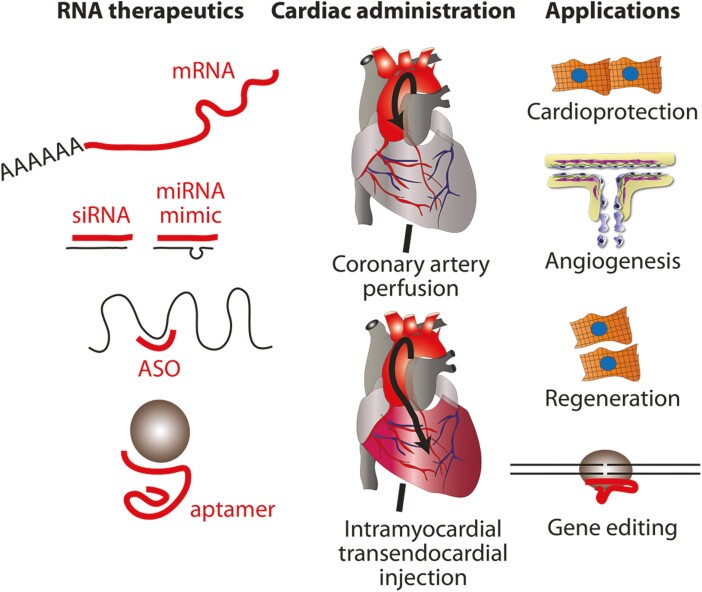

**Significance Statement**

RNA therapies hold a great promise to treat several cardiac conditions, including stimulation of cardiac regeneration. This review details the main possible applications of RNA therapies and defines the technological and biological barriers that still need to be overcome for these therapies to become clinically effective.

## Introduction

The repair of damaged hearts remains a holy grail of clinical medicine. Advances in early revascularization after myocardial infarction, the development of devices sustaining the failing myocardium and better clinical management have significantly improved the outcome of heart failure. Still, no drug or treatment is available to protect the heart from damage or promote its regeneration. Neither are any effective treatments available to stimulate new cardiac blood vessel formation. All current medical therapies for myocardial infarction and heart failure essentially aim to sustain the function of the survived myocardium and are based on the combination of small molecules.^.1^

Over the last couple of decades, there has been a growing appreciation that a large set of noncoding RNAs (ncRNAs) regulate virtually all biological functions of the cells. According to a recent consensus statement,^2^ these ncRNAs can be classified into one of three classes. The first class includes small RNAs, usually less than 50 nucleotides (nt), of which the most relevant ones are the small RNAs involved in the RNA interference (RNAi) pathway (in particular, the microRNAs or miRNAs; over 2500 mature sequences encoded by the humangenome^3^). A second class includes a heterogenous series of RNAs, typically between 50 and 500 nt, which include RNA polymerase III (Pol III) transcripts such as tRNAs or ribosomal 5S rRNAs, Y RNAs, small nuclear RNAs (snRNAs) and intron-derived small nucleolar RNAs (snoRNAs). A third class includes long noncoding RNAs (lncRNAs), which are more than 500 nt in length and are mostly generated by Pol II. These lncRNAs are estimated to be well in excess of 100 000 different molecules in humans.^4^lncRNAs also include circular RNAs (circRNAs) generated by backsplicing of coding and noncoding RNAs.^5^

Together with protein-coding mRNAs, these ncRNA molecules participate in a very broad range of cellular processes, including in all cells of the cardiovascular system. All these processes are thus amenable to regulation by either exogenously administering RNA molecules or by targeting the endogenous RNAs using complementary small RNA segments. The RNA therapeutics field has expanded quicky over the last 5 years for several noncardiac conditions. To date (March 2023), 14 ncRNAs—9 antisense oligonucleotides (ASOs) and 5 short interfering RNAs (siRNAs) —have already reached clinical approval,^6^ together with 2 mRNA-based COVID-19 vaccines.

To what extent and how quickly can we also envisage the development of a new class of RNA therapeutics for cardiac repair and regeneration, which could overcome the need for developing stem cell-based cell transplantation approaches? And what are the hurdles that still need to be overcome to develop such RNA therapies? Here, I will briefly discuss the main foreseeable applications in this area and highlight the barriers that still need to be overcome for effective clinical application.

## Types of RNA Therapeutics

**Figure 1.**
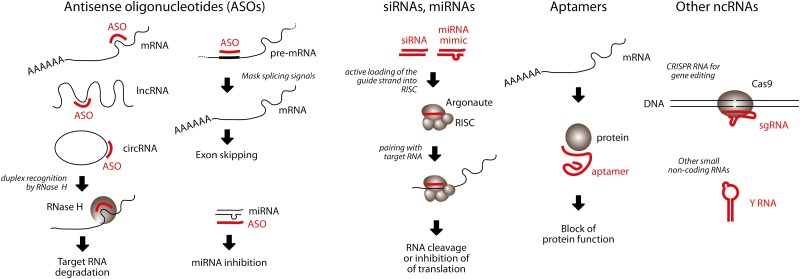
Schematic representation of the main classes of small, noncoding RNA therapeutics. The first category of small, noncoding RNAs (ncRNAs) includes antisense oligonucleotides (ASOs), which can target both coding and noncoding RNAs (the latter can be long noncoding RNAs (lncRNAs), circular RNAs (circRNAs), or microRNAs (miRNAs). In the cases of mRNAs, circRNAs, and lncRNAs, ASOs can drive the degradation of the RNA molecule; in the case of miRNAs, they usually provide steric inhibition. ASOs targeted to pre-mRNAs in the nucleus can be used to control splicing, in particular for exon skipping purposes. A second category includes RNA interference (RNAi) therapeutics, in particular short interfering RNAs (siRNAs) or endogenous miRNA mimicking molecules (miRNA mimics). A fourth category includes RNA molecules that interact with proteins by virtue of their 3D structure (aptamers). Finally, another class of small ncRNAs includes guide RNAs for CRISPR/Cas9 applications (in particular, single guide RNAs—sgRNAs) and other noncoding small RNAs such as Y RNAs.

The types of RNA therapeutics can essentially be divided into 2 categories according to their length (long and short RNAs), as these 2 kinds of molecules not only have different biological properties but also pose different challenges in terms of therapeutic administration. Long RNAs can either code for protein, acting as mRNAs, or not (lncRNAs). Short therapeutic RNAs are all noncoding and can in turn be classified into 4 different classes (Fig. 1). The first class is antisense oligonucleotides (ASOs), which are highly modified RNA species that target long RNAs. The targets of ASOs can be mRNAs, lncRNAs, or circRNAs to induce their degradation, pre-mRNAs to inhibit their processing, or microRNAs -miRNAs—to inhibit their function. A second class of ncRNAs are molecules that intercept the cellular RNA interference (RNAi) pathway (miRNAs or siRNAs).^6^ A third class of molecules includes aptamers, namely RNAs that bind a specific target, which can be either a biological or a small chemical molecule, by virtue of their 3D conformation^7^ Finally, a fourth class of ncRNAs includes a heterogenous group of small RNAs with different functions. Among these molecules, there are CRISPR RNAs to guide Cas molecules onto specific DNA targets and other cellular regulatory RNAs, such as Y RNAs.^8^

## RNA Therapies for Cardiac Repair and Regeneration

Applications of RNA therapies for cardiac repair and regeneration can be foreseen in at least 4 areas. These include protection from acute myocardial damage (cardioprotection), induction of new blood vessel formation (angiogenesis), stimulation of cardiomyocyte proliferation (cardiac regeneration) and correction of cardiac inherited conditions (gene editing) (Table 1). To date, there are no approved RNA therapies for any of these conditions. RNA therapies are also being developed for the treatment of chronic heart failure, with the purpose of improving cardiac function. This requires chronic administration, and I will not treat this topic further here.

**Table 1.** Current and potential applications of RNA therapies for cardiac repair and regeneration (selected examples). Reference to published work using RNA administration is indicated. Applications without citations are potential.

Application	RNA class	Therapeutic RNA or antisense target	Delivery method	Most advanced stage of application	References
Cardioprotection	ASO	miR-92a, miR-34, and miR-124	Naked	Small animals	^12-14^
	miRNA mimic	miR-19a/19b	Lipofection	Small animals	^15^
	mRNA	Cardioprotective proteins (eg, Chrdl1 and Fam3c)	LNPs		
Neoangiogenesis	mRNA	VEGF-A	Modified RNA injection	Clinical trial	^28^
Regeneration	shRNA	SAV1	AAV vector	Large animals	^32^
	Pri-miRNA gene	miR-199a, miR-590, miR-294, and miR-19a/19b	AAV vectors	Large animals	^15, 34-36^
	miRNA mimic	miR-199a-3p, miR-590-3p, miR-19a/19b, miR302b/c, and miR-708	Lipofection	Small animals	^15, 37-40^
	mRNA	PKM2	Modified RNA injection	Large animals	^41^
		Extracellular factors (eg, Fstl1, NRG1, and agrin)			
		Cell cycle regulators (E2F, cyclin A, and cyclin D)			
		Reprogramming factors for ES cells (eg, Oct4, Sox2, Klf4, and c-Myc)			
		Reprogramming factors for cardiomyocytes (eg, Gata4, Mef2c, and Tbx5)			
Correction of cardiac genetic defects	mRNAs, sgRNAs	CRISPR RNA and mRNA for Cas editors	LNPs		
	ASO	Exon skipping	Naked	Small animals	^46, 47^
	RNA	RNA trans-splicing	AAV vector	Small animals	^49^

Abbreviations: DCM, dilated cardiomyopathyl LNPs, lipid nanoparticles;

### Cardioprotection

Loss of cardiomyocytes accompanies virtually all conditions leading to heart failure. This can be dramatic during myocardial infarction, when the heart can lose as many as 25% of the 2-4 billion cells that are present in the left ventricle,^9^ but also occurs more chronically in several other pathological conditions, ranging from disorders of cardiac overload (such as hypertensive cardiomyopathy or aortic stenosis) to cancer chemotherapy. As the formation of new cardiomyocytes does not occur at a clinically significant rate after birth, the goal of sparing cardiomyocytes from death is of paramount importance.

Multiple studies in the last couple of decades have focused on the events that occur immediately after ischemia and reperfusion, showing that brief episodes of coronary occlusion/reperfusion preceding or following infarction (ischaemic pre- and post-conditioning, respectively) can reduce infarct size. Translation of these observations to the clinic, however, has failed so far (reviewed in ref.^10^). Of note, the need for cardioprotection is not necessarily restricted to the time immediately following ischemia–reperfusion, as a vast number of cardiomyocytes can be saved in the first 2-3 days after the event in the so-called area at risk, which is significantly larger than the actual infart region.^11^Experimental evidence shows that several ncRNAs can exert an acute cardioprotective effect. Examples include antisense inhibition of miR-92a,^12^ miR-34^13^ or miR-124,^14^ administration of miR-19a/19b mimic,^15^ or inhibition of the cardiac-specific lncRNA ZFAS1.^16^

The potential of ncRNA therapies for acute cardioprotection is also indirectly supported by the observation that several cell types originally injected into the heart for their presumed regenerative properties do not lead to cardiac regeneration but still exert a transient cardioprotective activity through a paracrine effect, which also involves the production of ncRNA-rich extracellular vesicles (EVs; reviewed in refs^17,18^:). MicroRNA-loaded EVs with a cardioprotective effect are also spontaneously released after myocardial infarction by platelets or different cells in the heart, in addition to being abundant in the circulation.^19^

A few proteins have also been described to exert acute and chronic cardioprotective activity. For example, recent work from my own laboratory has identified three novel cardioprotective proteins (Chrdl1, Fam3c, and Fam3b) from an AAV library of 1200 factors that were selected in vivo for cardioprotection in infarcted mice.^20^ As an alternative to the use of recombinant proteins, the mRNA coding for these factors can be administered to the heart immediately after myocardial infarction. A similar application can also be conceived for cardioprotective lncRNAs (for example, lncCIRBIL^21^) or circRNAs (for example, CircFndc3b^22^).

The possible modalities for the delivery of small, ncRNA, or large RNA therapeutics to the heart for cardioprotection and the problems that still need to be overcome will be discussed later, as these are not significantly different from those encountered by the same types of molecules for other cardiac applications.

### Therapeutic Angiogenesis

The induction of new blood vessel formation to counteract cardiac ischemia by gene transfer is one of the still unaccomplished goals of gene therapy. Angiogenesis is known to be controlled by a series of growth factors that act in concert to activate both endothelial and mural cells. For endothelial cells, these factors include, among several others, the VEGF family members with their different splicing isoforms,^23^ some of the fibroblast growth factor (FGF) family members^24^ and the hepatocyte growth factor (HFG).^25^ All of these have been proven effective in driving angiogenesis in both small and large animal models.

Taken together, these observations suggest that the delivery of the mRNA coding for these angiogenic factors, either alone or in combination, could induce therapeutic angiogenesis in ischemic hearts, as RNA gene transfer is more effective than plasmid transfection and less inflammatory than adenoviral vectors, which are the 2 modalities used by gene therapy clinical trials so far.^26^ Based on the efficacy of a naked, modified mRNA (modRNA) coding for VEGF-A in mice^27^ and the results of preliminary safety experimentation, a phase II clinical trial was started based on the injection of this modRNA in the myocardium of patients receiving elective coronary artery bypass surgery.^28^ Earlier in the summer of 2022, however, this trial was removed from the company’s phase II development pipeline (https://www.astrazeneca.com/investor-relations/results-and-presentations.html). The problems that still need to be overcome for effective therapeutic angiogenesis will be discussed later.

### Cardiac Regeneration

Cardiac regeneration in other species, including fish and amphibians, is not sustained by the expansion and differentiation of stem cells, but by the proliferation of already committed cardiomyocytes.^29,30^ Thus, an appealing possibility to induce cardiac regeneration is the stimulation of endogenous cardiomyocyte proliferation using gene or RNA transfer. An obvious problem, however, is how to identify the most effective therapeutic molecule.

At least 3 main signal transduction pathways sustain cardiomyocyte proliferation during embryonic, fetal and early neonatal life, and in principle can be stimulated by genetic manipulation. These are the Wnt/β-catenin, the Notch, and the Hippo pathways (reviewed in Ref.^31^). The positive effectors of these pathways (β-catenin, Notch intracellular domain, and YAP) are all activators of cardiomyocyte proliferation, and thus either these factors themselves or the regulatory pathways leading to their activation can in principle be leveraged for cardiac regeneration through mRNA or siRNA delivery of the activators and repressors respectively. Work from the Martin laboratory has already shown that the injection of an AAV vector expressing an shRNA against SAV1, a cofactor of the YAP inhibitory kinase MST1/2 (Hippo in Drosophila), leads to cardiomyocyte proliferation and cardiac regeneration in pigs.^32^ A similar application can be developed based on naked siRNA injection.

Multiple evidence over the last 10 years, including the results of systematic screenings, has shown that several miRNAs regulate the rate of cardiomyocyte proliferation.^33^These miRNAs include members of families that are highly expressed in embryonic stem cells (eg, the miR-302-367 and miR-miR-290 families), miRNAs that also regulate the proliferation of cancer cells (eg, the miR-17-92 and miR-106b-25 clusters), along with individual miRNAs identified through systematic screenings (eg, miR-590-3p, miR-199a-3p, and miR-1825). Other miRNAs are instead expressed in nonreplicating cardiomyocytes and have an inhibitory role (eg, the let-7 and miR-15 family miRNAs). While the inhibition of these suppressive miRNAs using ASOs has a limited role in therapeutic cardiac regeneration, several laboratories have reported on the regenerative effect of the positive administration of pro-proliferative miRNAs. After myocardial infarction in mice, miR-199a or miR-590a,^34^ miR-294, a member of the miR-302 superfamily,^35^ and the miR-17 ~ 92 cluster member miR-19a/19b,^15^ in all cases expressed using AAV vectors, led to cardiac regeneration and restitution of cardiac function.

The permanent expression of pro-proliferative miRNAs from AAV vectors can be problematic for at least 2 reasons. First, expression of miRNA cannot be controlled over time and, second, both the desired miRNA strand and the miRNA produced from the complementary strand (the 5p and 3p miRNAs) can be generated within the transduced cells. This can lead to undesired effects over time, as recently proven by one of our experimentations in pigs using miR-199a.^36^ In all these conditions, the possibility of delivering synthetic ncRNAs remains appealing. Available evidence already shows that, in mice, the single intramyocardial injection of miR-199a-3p or miR-590-3p,^37^ miR-19a/19b,^15^ miR302b/c,^38,39^ miR-19a/19b^15^ or miR-708^40^ mimics is effective for cardiac regeneration. A challenge remains how to formulate these mRNAs in clinically approvable preparations and prove their efficacy in larger animals.

Some extracellular and intracellular proteins were also proven to stimulate cardiomyocyte proliferation. These include a few growth factors (eg, interleukin-6, PDGF, members of the FGF family, follistatin-like-1, and neuregulin-1), the extracellular matrix protein agrin, cell cycle regulators such as E2F family transcription factor, or cyclins A2, D1, and D2 (for specific references cf. Ref.^33^) and the metabolic enzyme PKM2.^41^ Additionally, the transient activation of the 4 reprogramming factors Oct4, Sox2, Klf4, and c-Myc (OSKM), used to generate iPS cells, can partially dedifferentiate cardiomyocyte and stimulate their proliferation,^42^ while the administration of other transcription factors (eg, Gata4, Mef2c, and Tbx5—GMT) can directly reprogram fibroblasts into cardiomyocytes.^43,44^

For all these positively acting proteins, the delivery of their in vitro transcribed, modified mRNAs to the heart could be conceived as a mean to stimulate regeneration. The L. Zangi laboratory has already provided evidence that the administration of a modified mRNA for PKM2 can induce regeneration after myocardial infarction in both mice^41^ and pigs (together with the J. Zhang group; American Heart Association Scientific Sessions 2022). Besides the problems of delivery, the main issue for such applications is the relatively short half-life of long RNAs, which, in contrast to small ncRNAs that can persist for over 1 week in an active form,^37^ have a much shorter duration.^45^ In addition, activation of the innate immune response is usually significant using long RNAs, despite the chemical modifications that are introduced during in vitro transcription.^45^

### Cardiac Gene Editing

Several inherited cardiac conditions, including structural and functional genetic defects of cardiomyocytes (cardiomyopathies and inherited arrhythmia syndromes) are, in principle, amenable to be treated using RNA therapies. Noncoding RNA-based treatments have already been tested in some of the dilated cardiomyopathy preclinical models. These include ASOs for exon skipping in titin^46^ and phospholamban^47^ cardiomyopathies, delivery of miR-669a as a gene modifier for sarcoglycan dysfunction^48^ or pre-trans-spliced RNA molecules that induce trans-splicing of lamin A/C^49^).

The CRISPR/Cas9 gene editing technologies now offer exciting new possibilities for direct DNA modification. The current standard for delivering a gene editing suite (including the editor, its guide RNA, and a template DNA for homology-directed repair) is by using AAV vectors. These vectors, however, are not devoid of problems. They have a limited cloning capacity, which prompts for the identification or generation of smaller endonucleases, require high viral titres which can lead to adverse events, and determine indefinite persistence of the endonuclease in the transduced cells, which is not desirable for reasons of toxicity and immunoreactivity. The possibility of delivering the endonuclease mRNA and guide RNA as in vitro transcribed molecules hold the potential to overcome all these limitations. Multiple preclinical studies point to the feasibility of administering these RNAs in the context of lipid nanoparticles (LNPs; reviewed in ref.^50^). A clinical trial for the treatment of transthyretin amyloidosis^51^ by knocking out the mutated gene in the liver has already generated interim positive results (American Heart Association Scientific Sessions 2022).

While these results reinforce the conclusion that gene editing can be achieved with nonviral delivery of the gene editing suite RNAs, the problems that need to be overcome to make this effective in the adult heart remain important and will be discussed in the next session.

## The Long and Winding Road Towards Clinical Application

RNA therapies for cardiac application remain exciting, but a series of technological problems need to be solved and, for some applications, a better understanding of the underlying biology is still needed. These issues are summarized here.

### The Problem of RNA Delivery

Nucleic acids are charged, hydrophilic and sensitive to nucleases, which are all obstacles to their effective administration. For ASOs, the introduction of chemical modifications can increase stability and pharmacological properties.^52,53^ In contrast to ASOs, miRNA mimics and siRNAs tolerate fewer chemical modifications, especially in their guide strand. Still, in vivo persistence of these molecules can also be extended. For example, inclisiran, a clinically approved siRNA against PCSK9 for the treatment of familial hypercholesterolemia, is initially administered by a single subcutaneous injection, repeated after 3 months and then every 6 months. Chemical modifications can also be introduced in mRNAs during in vitro transcription. A common strategy for all current applications is the substitution of uridine with one of its analogs (eg, pseudouridine) that blunt activation of the innate immune response, and the co-transcriptional capping with the 5ʹ end Cap1 structure, which further helps suppress immunogenicity.^54,55^

A major problem remains the cellular uptake of these molecules, given the negative charge of nucleic acids. While short, single-stranded ASOs with a phosphorothioate backbone can cross a biological membrane when delivered as naked molecules,^56^ natural nucleic acids with a phosphodiester backbone require either conjugation with a ligand or delivery through a carrier molecule. The only ligand for ncRNAs that have progressed towards clinical application for targeting the liver is a trimer of Nacetylgalactosamine (GalNAc), which binds the cellular asialoglycoprotein receptor (ASGPR; reviewed in ref.^57^). No such ligand still exists for the heart.

In vitro transcribed, modified mRNA was reported to enter cardiomyocytes as a naked molecule when administered by intramyocardial injection in a sucrose–citrate buffer.^41,58^ Most RNA applications, however, take advantage of the possibility of delivering nucleic acids using formulations based on polymers, lipids, or other nanosized particles. A specific type of formulation is lipid nanoparticles (LNPs) obtained by the Stable Nucleic Acid Lipid Particle (SNALP) technology. This originally dates back to the late 1990s and has been progressively improved in the early 2000s^59,60^ to become very popular after the COVID-19 vaccines. SNALP LNPs are 60-120 nm diameter particles in which the RNA is surrounded by a lipid bilayer formed of 4 lipids, of which one is an ionisable lipid that binds the RNA in acidic conditions to then become neutral at physiological pH (reviewed in ref.^61^). Besides the 2 COVID-19 vaccines, the SNALP technology was instrumental for the first siRNA product to be approved in the clinic, patisiran for transthyretin amyloidosis in 2018,^62^ and the above-mentioned in vivo gene editing clinical trial for the same condition.^51^

### The Problem of Targeting

While SNALP LNPs continue to be very promising and additional delivery methods are constantly developed, it is becoming quite clear that most systemically administered, nucleic acid-containing nanoparticles are phagocytosed by mononuclear cells in the liver, spleen, lymph nodes, and bone marrow or end up in the liver, while only a small fraction is retained in the heart. A recent study showed that the systemic injection of LNPs containing the DLin-MC3-DMA ionisable lipid, the same as patisiran, transfected the heart with an efficiency that was several orders of magnitude lower than that of the liver.^63^ Cardiac uptake was significantly increased after ischemia/reperfusion, but transfection was mainly of noncardiomyocyte cells in the infarcted area.

Therefore, a holy grail in the field of nonviral, cardiac RNA delivery is to find ligands that would permit specific targeting of cardiomyocytes, similar to the above-mentioned GalNac sugar for uptake in hepatocytes. A few cardiac-specific peptides have been identified through phage display panning techniques, or are ligands for cardiac cell-specific receptors, or antibodies against cardiomyocyte or endothelial cell receptors (cf. Ref.^6^ for specific references). Methods can also be developed to enrich for a desired miRNA into engineered EVs^64^ and to include, within the EV membrane, specific ligands to achieve tissue targeting.^65^

Despite these technologies are collectively quite promising in cell culture and mice, they appear still immature for clinical application, with liver detargeting remaining the main unsolved problem.

### Direct Administration of RNA Medicines to the Heart

While waiting for an effective targeting system to be developed, cardiac applications can leverage the possibility of administering molecules directly to the heart. .The intramyocardial injection can be performed through surgical access using a mini-thoracotomy followed by trans-epicardial delivery, or during coronary bypass surgery. In a significantly less invasive manner, an intramyocardial injection can be performed by trans-endocardial delivery from an intraventricular catheter inserted percutaneously to reach the cavity of the left ventricle. Several of these catheters have been tested and approved for human application (reviewed in ref.^26^). The site of injection in the left ventricle muscle can be identified preoperatively by imaging techniques and confirmed during the procedure by electromechanical mapping or imaging.

An even more straightforward and less invasive application is intracoronary administration, which is commonly performed for percutaneous coronary intervention (PCI; balloon angioplasty). This type of administration has already been used for cell delivery in over 30 clinical studies.^66,67^ A main issue with coronary delivery is that the administered RNA nanoparticles compete with the coronary flow, which can rapidly flush them out of the coronary circulation. This can be compensated by infusion during a sub-occlusive procedure, the use of agents that increase permeability or by retrograde, instead of anterograde, RNA nanoparticle administration. When the aim is to induce acute cardioprotection or regeneration after myocardial infarction, applications can take advantage of the so-called enhanced vascular permeability and retention (EPR) effect, by which nanosized particles can extravasate from the vasculature and accumulate in tissue thanks to the generation of large fenestrations between endothelial cells due to inflammation.^68^ The EPR effect fades after approximately 48 h and thus provides a time window that is sufficiently long for therapeutic administration.

### The Problem of Better Understanding Cardiac Biology

The success of RNA therapies for the heart not only requires a series of technical problems to be solved but also that effective RNA molecules are identified. This is well exemplified by the lack of success of all the clinical studies performed so far for therapeutic angiogenesis, and in particular of those using VEGF-A. Our current information indicates that: First, angiogenesis requires at least 30 different factors to lead to the formation of functional neo-vessels, while the vasculature formed in response to VEGF-A alone is leaky and not functional.^69^ Second, in experimental models VEGF-A requires at least 2-3 weeks of continuous expression before a stable vasculature is generated.^70,71^ Instead, the VEGF-A modified mRNA has a terminal half-life of 28 h after intracardiac administration.^72^ Third, the adult heart is a poorly angiogenic environment, in which new blood vessel formation is suppressed.^73^ Taken together, these observations reduce the surprise that the transient delivery of VEGF-A is poorly effective and prompt a better understanding of the underlying biology of the cardiac endothelium for effective therapies to be eventually developed.

A similar need for better basic understanding is universal in virtually all fields of cardiac biology. Another example derives from familial cardiomyopathies and inherited defects of the cardiac rhythm, which pose formidable problems to gene treatments. The cDNAs of several of the genes causing these conditions are too large to fit into AAV vectors, hence the recessive forms cannot be easily addressed by replacement gene therapy. Then, several of the mutated genes, in particular those coding for ion channels or desmosomal proteins, have a dominant or codominant inheritance, which would be amenable to gene disruption using CRISPR/Cas9 followed by repair by the cellular Non-Homologous-End-Joining (NHEJ) machinery. However, allele discrimination is poor single-point mutations, as is often the case, can lead to allele insufficiency. In these instances, precise gene correction of the mutated allele would be ideal, but homology direct repair is inefficient in cardiomyocytes. A better understanding of how to induce the homologous recombination machinery in postmitotic cells is thus required for the development of effective RNA treatments.

## Conclusions

The possibility of developing RNA-based medicines for cardiac repair and regeneration remains exciting and has disruptive potential. However, the successful generation of such a new class of drugs depends on our capacity to overcome the current technical obstacles in delivering nucleic acids to the heart and in better identification of the most effective molecules and their targets.

## Data Availability

No new data were generated or analyzed in support of this research.
